# Supplementary material for: A Systematic Two-Sample Mendelian Randomization Analysis Identifies Shared Genetic Origin of Endometriosis and Associated Phenotypes
Source: Life (Basel). 2021 Jan 3;11(1):24. doi: 10.3390/life11010024 (PMC7824623; doi:10.3390/life11010024)
Supplement: Supplementary file 1 [file life-11-00024-s001.zip › Garitazelaia-et-al_Supplementary-files_RESUBMISSION_2020-12-29/Garitazelaia-et-al_Figure-S1.pptx]

## Slide 1
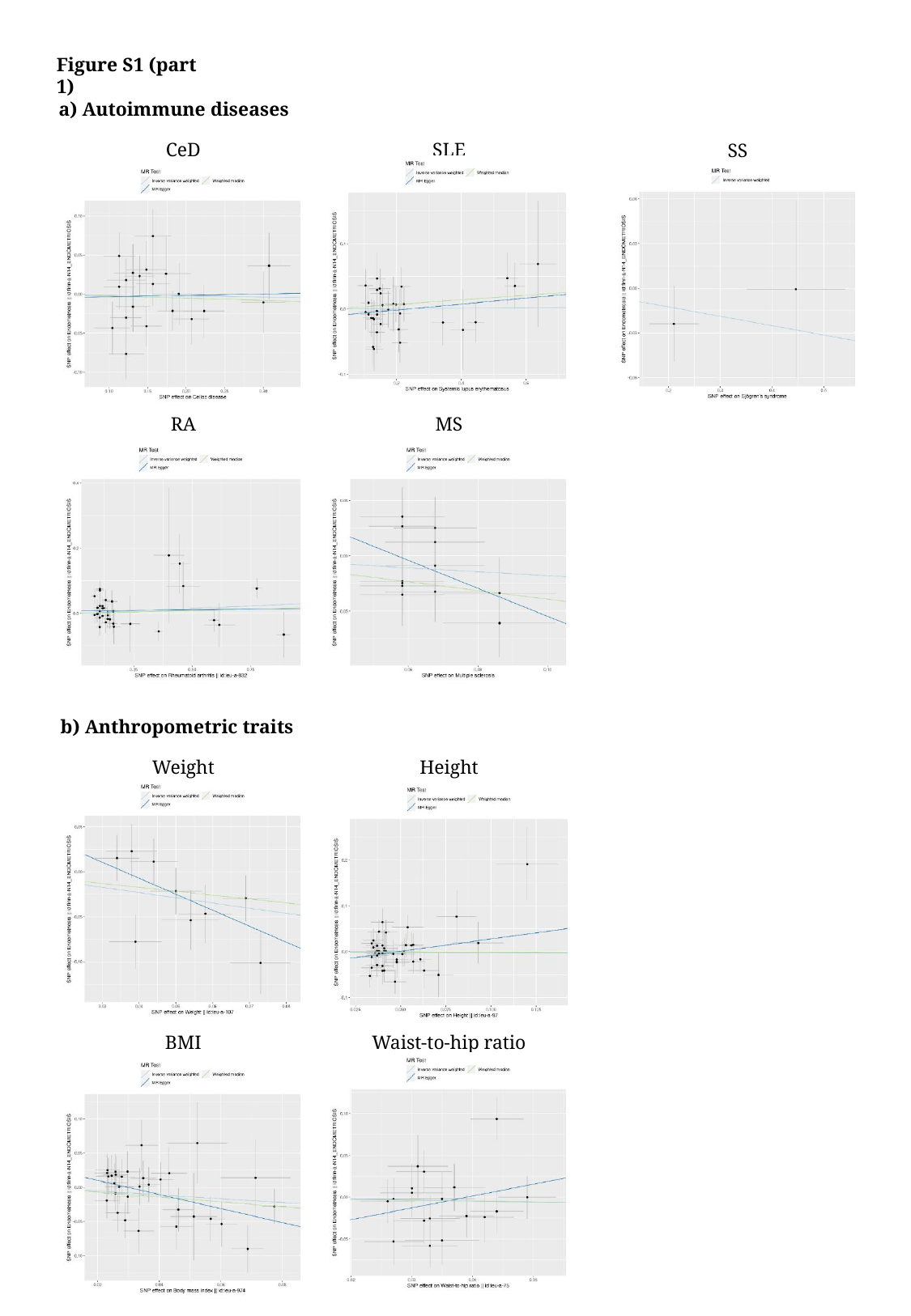

Figure S1 (part 1)
a) Autoimmune diseases
CeD
SLE
SS
RA
MS
b) Anthropometric traits
Weight
Height
BMI
Waist-to-hip ratio

## Slide 2
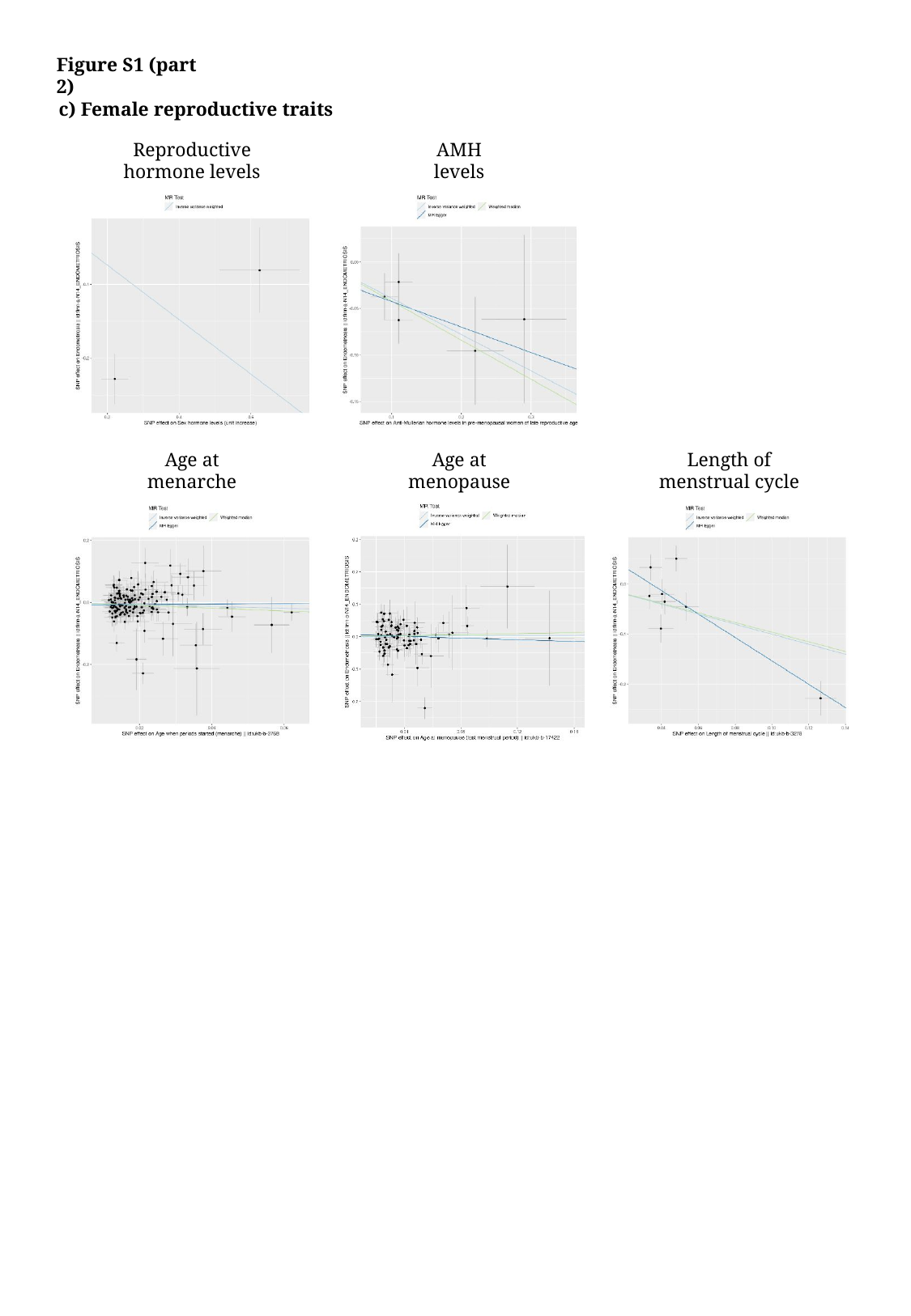

Figure S1 (part 2)
c) Female reproductive traits
Reproductive hormone levels
AMH
levels
Age at
menarche
Age at
menopause
Length of menstrual cycle
